# Social inhibition and behavioural flexibility when the context changes: a comparison across six primate species

**DOI:** 10.1038/s41598-018-21496-6

**Published:** 2018-02-15

**Authors:** Federica Amici, Josep Call, Julia Watzek, Sarah Brosnan, Filippo Aureli

**Affiliations:** 1 Institute of Biology, Faculty of Life Sciences, University of Leipzig, 04103 Leipzig, Germany; 2 Department of Primatology, Jr. Research Group “Primate Kin Selection”, Max Planck Institute for Evolutionary Anthropology, 04103 Leipzig, Germany; 3 Department of Comparative and Developmental Psychology, Max Planck Institute for Evolutionary Anthropology, 04103 Leipzig, Germany; 4 School of Psychology and Neuroscience, University of St Andrews, St Andrews, KY16 9JP Scotland UK; 5 Department of Psychology and Language Research Center, Georgia State University, P.O. Box 5010, Atlanta, GA USA; 6 Center for Behavioural Neuroscience and Neuroscience Institute, Georgia State University, P.O. Box, 5030 Atlanta, GA USA; 7 Instituto de Neuroetologia, Universidad Veracruzana, 91190 Xalapa, Veracruz Mexico; 8 Research Centre in Evolutionary Anthropology and Palaeoecology, Liverpool John Moores University, L3 5UA Liverpool, UK

## Abstract

The ability to inhibit previously employed strategies and flexibly adjust behavioural responses to external conditions may be critical for individual survival. However, it is unclear which factors predict their distribution across species. Here, we investigated social inhibition and behavioural flexibility in six primate species (chimpanzees, bonobos, orangutans, gorillas, capuchin monkeys and spider monkeys) differing in terms of phylogenetic relatedness, foraging ecology and social organization. Depending on the social context, individuals could maximize their food intake by inhibiting the selection of a larger food reward in one condition (i.e. inhibition), but not in others, which required them to flexibly switching strategies across conditions (i.e. behavioural flexibility). Overall, our study revealed inter-specific differences in social inhibition and behavioural flexibility, which partially reflected differences in fission-fusion dynamics. In particular, orangutans and chimpanzees showed the highest level of inhibitory skills, while gorillas and capuchin monkeys showed the lowest one. In terms of behavioural flexibility, orangutans and spider monkeys were the best performers, while bonobos and capuchin monkeys were the worst ones. These results contribute to our understanding that inhibition and behavioural flexibility may be linked in more complex ways than usually thought, although both abilities play a crucial role in efficient problem solving.

## Introduction

Flexibility allows animals to modify their behaviour based on brief, limited experience, responding rapidly to subtle variations in consequences or context^1^. The ability to flexibly adjust behavioural responses depending on the external conditions might critically affect the survival and fitness of individuals in complex dynamic environments^2–9^, and may significantly differ across species^7^. In primates, for instance, inter-specific differences in behavioural flexibility have been linked to inter-specific differences in general cognitive skills^10–12^. Although conserved learning processes and perseverance might be sufficient to solve novel problems^7^, enhanced cognitive skills, such as analogical reasoning and inhibition, might indeed facilitate the emergence of behavioural flexibility^6,13,14^. Additionally, when the context is changing, flexibly adopting a new behavioural strategy may imply the inhibition of previously employed strategies^12,15^. Therefore, inhibition might play a crucial role in the ability to flexibly solve novel problems.

Species’ ability to flexibly solve problems may be related to their socio-ecological characteristics, which in turn may be associated with their cognitive skills. Indeed, measures of cognitive abilities and learning correlate with problem solving skills in both birds and primates^16^. Moreover, a higher degree of frugivory, which requires individuals to rely on resources that vary in their spatiotemporal distribution, appears to be linked to higher cognitive demands and ultimately to a higher ability to flexibly solve problems^17–19^. Similarly, primate species with a more diverse, omnivorous diet are known to be less neophobic^20,21^ and to have better inhibitory skills^22^, and they might thus be more successful at flexibly solving novel problems. Flexible problem solving may also be linked to social characteristics. High behavioural flexibility, for instance, may be needed in frequently changing social environments, like those typical of species characterized by high levels of fission-fusion dynamics (i.e. frequent formation of subgroups of variable size and composition^23^). Under these circumstances, it may be beneficial for individuals to flexibly switch among behavioural strategies depending on subgroup composition (e.g. attacking group members depending on the presence of coalitionary partners in the subgroup).

One approach to investigate behavioural flexibility, and the role of inhibition in the ability to flexibly solve novel problems, is the study of tactical deception^24–26^. When engaging in tactical deception, individuals have to assess the social context and wait for the right moment to act. In a typical experiment, subordinate individuals are shown how to access food and are then given the opportunity to retrieve it, either when a dominant group member can steal the food or after the dominant has left. Appropriately timing food retrieval dependent on the social context (i.e. presence and position of the dominant partner) is considered a form of tactical deception^25,27^. Indeed, refraining from eating when higher-ranking partners are close by requires individuals to assess the social context and respond accordingly, suppressing the prepotent response of eating when dominants are nearby (i.e. inhibition), but reaching for the food as soon as the dominants have left (i.e. behavioural flexibility). The occurrence of flexible problem solving in tactical deception has been clearly demonstrated in chimpanzees (*Pan troglodytes*)^25,27^, mangabeys (*Cercocebus torquatus*)^24^, Tonkean macaques (*Macaca tonkeana*)^28^, brown capuchin monkeys (*Sapajus [Cebus] apella*)^29,30^ and, to some degree, in lemurs (*Lemur catta*^31^, *Eulemur macaco*^32^, *E. fulvus*^33^). However, differences in the experimental procedures make it hard to draw conclusions about inter-specific differences.

To our knowledge, only two studies have directly compared levels of behavioural flexibility and social inhibition by using the same experimental procedure across more than one primate species. In one study, spider monkeys (*Ateles geoffroyi*), long-tailed macaques (*Macaca fascicularis*) and brown capuchin monkeys were tested in a tactical deception task^34^. All species were able to inhibit the retrieval of food when a dominant group member was in proximity, but spider monkeys were significantly more efficient than the other species at timing food retrieval contingently of the dominant’s departure, suggesting that high levels of fission-fusion dynamics may be linked to higher behavioural flexibility^34^. Using a different approach and humans as interacting partners, Reddy and colleagues^35^ have recently compared social inhibition in five lemur species with different socio-ecological characteristics. In this study, individuals were presented with identical food rewards by two experimenters, only one of whom allowed subjects to retrieve it. After being trained to approach the “generous” experimenter, subjects were offered a smaller food reward by the “generous” experimenter and a larger food reward by the “competitive” experimenter, and therefore had to suppress the prepotent response of reaching for the larger reward when the social context made it unobtainable. In this study, no inter-specific differences in social inhibition were found^35^.

To date it is still unclear how social inhibition and behavioural flexibility are distributed across primate species, and whether specific socio-ecological characteristics predict their distribution. In this study, we thus used a single experimental procedure to directly compare behavioural flexibility and social inhibition across six primate species, chimpanzees, bonobos (*Pan paniscus*), orangutans (*Pongo abelii*), gorillas (*Gorilla gorilla*), spider monkeys and brown capuchin monkeys. Specifically, we presented subjects with a smaller and a larger food reward, but we varied the position of the partner and of the food rewards. Depending on the social context (i.e. the position of the partner) and the food position, only one of the two rewards was accessible to the subject. Therefore, in order to maximize their food intake, subjects had to show both inhibition (i.e. selecting the smaller instead of the larger reward when the latter was not accessible) and behavioural flexibility (i.e. flexibly switching strategy across conditions, by selecting the larger reward when no inhibition was required).

The six species live in complex social systems, but differ in terms of phylogenetic relatedness, social organization and foraging ecology^22,36–42^, allowing us to assess the role of these factors in the distribution of behavioural flexibility and social inhibition (Table 1). In particular, we examined the role of (i) phylogenetic relatedness, predicting that apes would outperform monkeys, as phylogenetic relatedness could reflect more general differences in cognitive skills^43,44^; (ii) foraging ecology, predicting that the mostly folivorous gorillas would be outperformed by the relatively more frugivorous other five species, as the latter rely on more spatiotemporally distributed resources and might have thus overall evolved enhanced cognitive skills^22^; (iii) dietary breadth (i.e. number of dietary categories consumed by each species^22^), predicting that orangutans (having the largest dietary breadth) would outperform chimpanzees, gorillas, capuchin monkeys (having intermediate dietary breadth), who would outperform bonobos and spider monkeys (having the narrowest dietary breadth), as species with larger dietary breadth are more likely to exploit new food sources and might have thus overall evolved enhanced cognitive skills to better cope with this variation^22,45^; and (iv) the degree of fission-fusion dynamics, predicting that chimpanzees, bonobos, orangutans and spider monkeys, which experience higher degrees of fission-fusion dynamics, would outperform the more cohesive gorillas and capuchin monkeys, as species with higher spatiotemporal variation in subgroup size and composition could require higher inhibition (to better assess the new social situation before acting after fusion events) and behavioural flexibility (to better adjust to frequently changing social situations)^23^.

**Table 1:** Predicted relative performance of inhibition and behavioural flexibility skills based on phylogeny and socio-ecological factors compared to the results of our study.

Species	predictions				results	
	Phylogenetic relatedness	Foraging ecology	Dietary breadth	Fission-fusion	Inhib	Flexib
Chimpanzees	+/+	+/+	0/0	+/+	+	0
Bonobos	+/+	+/+	−/−	+/+	0	−
Orangutans	+/+	+/+	+/+	+/+	+	+
Gorillas	+/+	−/−	0/0	−/−	**−**	0
Spider monkeys	−/−	+/+	−/−	+/+	0	+
Capuchin monkeys	−/−	+/+	0/0	−/−	−	−

Confirmed predictions for inhibition/behavioural flexibility are highlighted in grey. “+” stands for a performance which was better than some of the other species, “−” for a performance which was worse than some of the other species, and “0” for an intermediate performance. “Inhib” stands for performance in ExpSR, a measure of inhibition, and “Flexib” for performance in ExpLR and ContSR, a measure of behavioural flexibility.

## Materials and Methods

### Subjects

We tested 6 spider monkeys (combined in 11 pairs) at the Animaya Zoo in Merida, Mexico, 8 capuchin monkeys (in 13 pairs) at the Language Research Center of Georgia State University, Atlanta, USA, and 6 chimpanzees (in 10 pairs), 5 bonobos (in 9 pairs), 5 orangutans (in 9 pairs) and 4 gorillas (in 12 pairs) at the Wolfgang Koehler Primate Research Center in the Leipzig Zoo, Germany. Subjects were of both sexes and various age classes (adults, subadults and juveniles^46,47^). Subjects were all born in captivity, except for the spider monkeys, who were born in the wild but were raised as pets before being rescued and brought to the zoo. Subjects were all housed in social groups with their conspecifics in enclosures with outdoor and indoor areas, and they were never deprived of food or water before or during the experiment. All of the subjects were familiar with being temporarily isolated in testing rooms and were tested by a familiar experimenter only after they were comfortable with the set-up. All subjects had previously participated in experimental tasks, but none of them had been previously tested with the experimental procedure of this study. Each subject was tested with one to three partners (see Table S1 in Supplementary Material).

### Procedure

**Figure 1:**
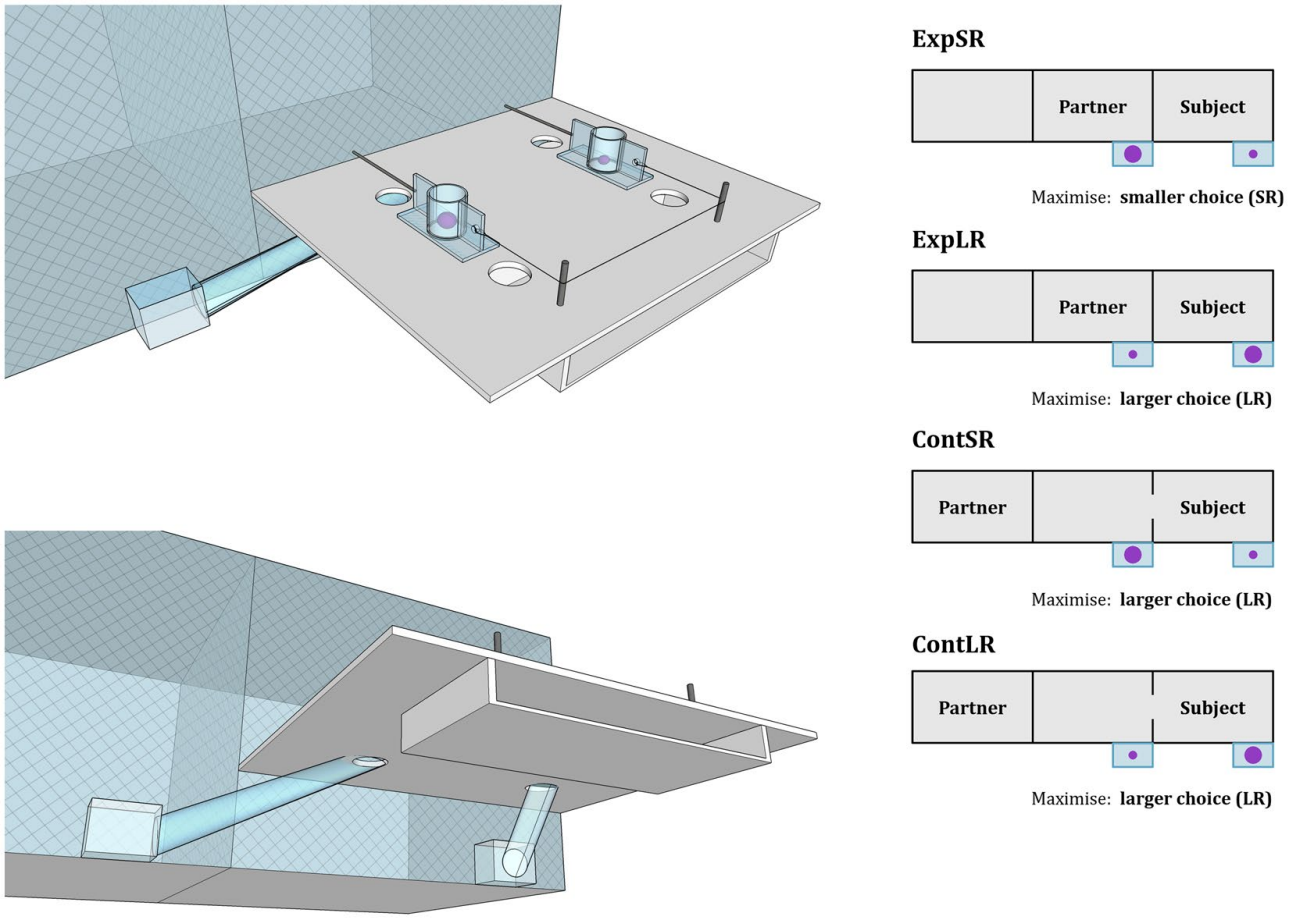
(**A**) Pictorial representation of the apparatus. (**B**) Setup for the trial types in the Experimental and Control condition. Due to differences in the species’ housing, for capuchin monkeys in the Control trials the partner was in a room adjacent to the back (rather than the side) of the other testing rooms.

All experimental protocols were approved by the Wolfgang Koehler Primate Research Center in the Leipzig Zoo, Germany, by the Animaya Zoo in Merida, Mexico, and by the Language Research Center of Georgia State University, Atlanta, USA, and the methods were carried out in accordance with the relevant guidelines and regulations. The apparatus consisted of a plastic table with two sliding platforms (Fig. 1). A transparent Plexiglas tube was attached to each of the two platforms. Platforms could be baited with food, which subjects could only reach after they selected one of the two platforms by pulling its handle. When the subject pulled one handle, the corresponding platform slid closer to the subject and the food placed on its surface rolled down the Plexiglas tube into a little plastic bowl, while the other platform slid back out of reach and the food on its surface disappeared into an opaque box underneath. The length of the tubes could vary, allowing us to place the plastic bowls in different positions depending on the conditions.

We administered three different phases. In the Training phase, we trained subjects to use the apparatus. Both plastic bowls were exclusively accessible to the subject. The Experimenter took a small grape and visibly baited only one of the two platforms (randomly changing the baited side and counterbalancing it within each session, with no side baited more than three times in a row). Successfully completing the Training phase (i.e. pulling the platform and retrieving the food from the bowls in at least 10 out of 12 trials in 2 consecutive sessions) was required before subjects proceeded to the next phase. Thirty-two out of 33 subjects completed the training after 2 sessions, and one subject after 3 sessions.

In the Preference phase, we tested whether subjects preferred a larger reward (LR) over a smaller reward (SR). Rewards were grapes selected from the same bunch, so that one was clearly smaller than the other one - approximately half the diameter. In this phase, both plastic bowls were exclusively accessible to the subject so that grapes could be accessed from both sides. The Experimenter took one LR and a SR and visibly baited each of the two platforms with one grape (changing and counterbalancing the side as above). Only subjects pulling the platform with LR in at least 10 out of 12 trials in 4 consecutive sessions moved on to the following phase. Thirty subjects passed the Preference phase after 4 sessions, two subjects after 5 and one subject after 6 sessions.

In the Test phase, we ran two different conditions. In the Experimental condition, we tested whether subjects could inhibit the selection of LR when the partner’s presence prevented them from retrieving the food. In this condition, a partner was present in the adjacent testing room (Fig. 1). One plastic bowl was exclusively accessible to the subject, while the other bowl was in the adjacent room and exclusively accessible to the partner. The Experimenter took a SR and a LR and visibly baited each of the two platforms in the same way as in the Preference phase. In this condition, however, subjects could only successfully retrieve the grape on the side opposite to the partner. If they pulled the platform on the partner’s side, the grape rolled into the corresponding bowl only within the partner’s reach. The optimal strategy was therefore to inhibit the selection of LR (i.e. choose the SR) when LR was on the partner’s side (ExpSR trials), and choose LR in the other trials (ExpLR trials).

In the Control condition, we followed exactly the same procedure as in the Experimental condition, but the partner was in another room with no access to any bowl (Fig. 1). In this condition, the bowls were positioned like in the Experimental condition, but the subject had exclusive access to both of them. In contrast to the Preference phase, therefore, the Control condition included the presence of a partner, but unlike in the Experimental condition, partners in the Control condition never had access to food. In the Control condition, the Experimenter could place the LR either on the side where the partner was in the corresponding Experimental condition (ContSR), or on the opposite empty side (ContLR trials). The optimal strategy was therefore to select LR in both ContSR and ContLR trials.

In this study, we therefore operationalized “inhibition” as the selection of SR in ExpSR trials, as individuals had to inhibit the prepotent motivation to select LR (see Model 1). However, subjects with little behavioural flexibility could show inhibition at the cost of wrongly generalizing the selection of SR to other contexts. In other words, subjects could learn to avoid one specific platform or the platform closest to the partner’s side in ExpSR, and then wrongly generalize this heuristic to ContSR and ExpLR trials (where food and partner, respectively, had the same position as in ExpSR trials). Therefore, we further operationalized “behavioural flexibility” as the selection of LR in ContSR and ExpLR trials, across all sessions (see Model 2), and across only those sessions in which subjects showed inhibition in at least 50% of the ExpSR trials (i.e. when subjects needed to switch strategy across trials; 50% was chosen as an arbitrary threshold for inhibition, as it implies that subjects inhibit in at least 3 trials, with possible effects on the other trials; see Model 3). In case of high behavioural flexibility, the selection of LR in these trials should not differ from that in ContLR trials, which did not pose a special challenge given that they had a different food and partner position from ExpSR.

For each dyad, we ran 6 sessions for the Experimental condition, and 6 sessions for the Control condition. Each session consisted of 12 trials. Within each session, we randomized the side with the LR, placing it 6 times on the partner’s side (in ExpSR and ContSR trials), and 6 times on the opposite empty side (in ExpLR and ContLR trials). For each dyad, we alternated the Experimental and Control sessions (starting with the Experimental condition with half of the subjects) and the side of the partner and of the inaccessible bowl (starting with the right bowl with half of the subjects).

### Statistical analyses

Analyses were conducted using logistic generalized linear mixed models^48^ with the lme4 package in R software (version 3.2.3^49^). Given that the dependent variable was binary (i.e. 0 for an incorrect choice and 1 for a correct choice), models were run with a binomial structure. All continuous variables were z-transformed to facilitate model convergence. We used a likelihood ratio test^50^ to compare full models (including test predictors and control predictors, which comprise fixed and random effects) with null models (including only control predictors). When full models differed significantly from null models, likelihood ratio tests were conducted to obtain the *p* values for each test predictor via single-term deletion^51^. An α-level of 0.05 was adopted for all tests (but trends were considered when the α-level was between 0.05 and 0.10), and post-hoc comparisons were conducted using Tukey corrections. Below we only report significant post-hoc tests and trends, but all post-hoc comparisons (including estimates and SE, and original data) can be found in Supplementary Material. No convergence issues were detected. In order to rule out collinearity, we used variance inflation factors (VIF^52^), which were good (maximum VIF across all models = 1.35).

Model 1 investigated how social inhibition varied across species and sessions in the ExpSR trials. In this model, the individual response in the ExpSR trials (1 correct, 0 incorrect) was the dependent variable, and session number (from 1 to 18, because subjects had up to three partners), species and their two-way interaction were the test predictors. As control predictors we entered the following fixed effects: subject’s sex, subject’s age class (juvenile, subadult or adult), partner position (right or left), performance in the Preference phase (i.e. mean percentage of trials in which the subject selected LR in the Preference phase) and trial number within each session (from 1 to 6), including all necessary random slopes. We further included subject and partner’s identities as random effects, to account for the non-independence of data points. Binomial tests further assessed if a species performed above chance in the first session in ExpSR trials.

Model 2 investigated how performance in ContSR, ContLR and ExpLR varied across species and trial type. In this model, the individual response in ContLR, ContSR and ExpLR (1 correct, 0 incorrect) was the dependent variable, and session number, trial type (ContSR, ContLR or ExpLR), species and their 2-way interaction were the test predictors. As control predictors we entered the following fixed effects: subject’s sex, subject’s age class, partner position, performance in the Preference phase and trial number, including all necessary random slopes; subject and partner’s identities were entered as random effects. Model 3 was identical to Model 2, but only included those sessions in which subjects made at least 50% correct choices in the ExpSR trials, in order to assess whether performance in ContLR, ContSR and ExpLR trials decreased when subjects effectively inhibited in ExpSR.

## Results

### Inhibition (ExpSR)

In Model 1, the comparison between the full model and the null model was significant (GLMM: *χ*^2^ = 49.41, df = 11, *p* < 0.001). We dropped the session*species interaction from the model as it did not significantly improve model fit (GLMM: *χ*^2^ = 3.54, df = 5, *p* = 0.618). There were significant species differences (GLMM: *χ*^2^ = 16.67, df = 5, *p* = 0.005; Table 2), and performance increased across sessions (estimate + SE = 0.36 + 0.04, *z* = 7.64, *p* < 0.001).

**Table 2:** Results of Model 1, in which the dependent variable was the selection of SR in ExpSR trials.

Test Category	*χ* ^2^	Df	*P*
**Species**	**16.67**	**5**	**0.005**
**Session**	**33.30**	**1**	**<0.001**
Subject’s sex	0.27	1	0.604
Subject’s age class	1.65	2	0.438
Partner’s position	1.28	1	0.257
Performance in Preference phase	0.93	1	0.334
**Trial number**	**29.91**	**1**	**<0.001**

Significant results in bold. See text for details on the test and control predictors.

**Figure 2:**
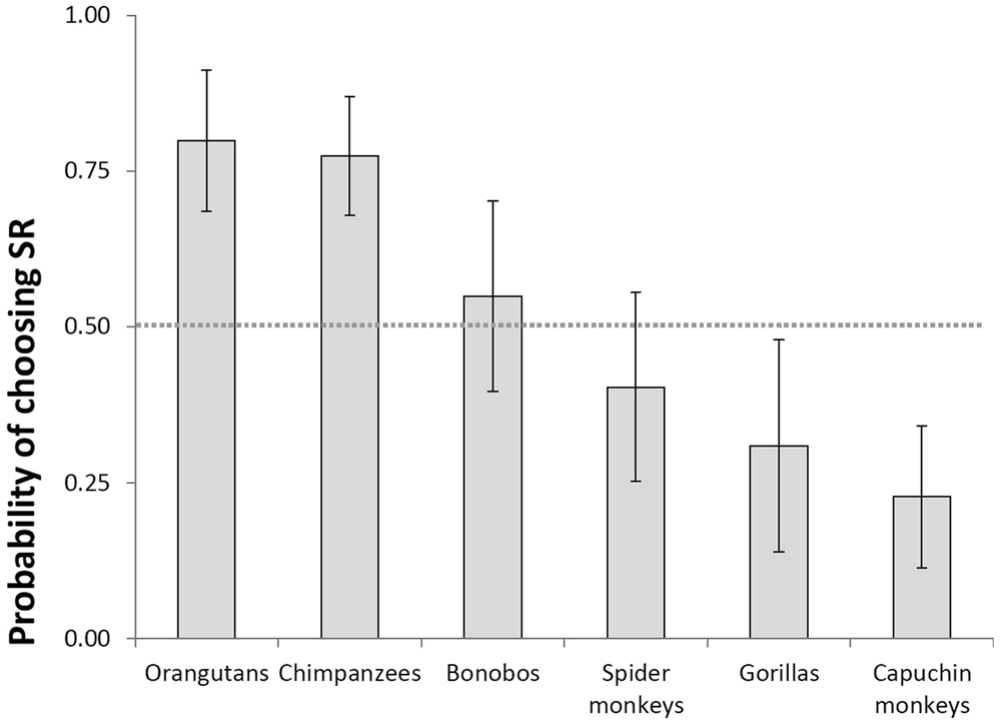
Mean (±SE) probability of making the correct choice (SR) in all ExpSR sessions for each species. Results are averaged over the levels of subject’s sex and age, and partner’s position (Model 1). The dotted line illustrates the probability of making a correct choice by chance.

Visual inspection of the data (Fig. 2) indicated that orangutans were the best performers, followed by chimpanzees, bonobos, spider monkeys, gorillas and capuchin monkeys. However, post-hoc tests for species confirmed only that capuchin monkeys performed significantly worse than orangutans (*z* = 3.43, *p* = 0.007) and chimpanzees (*z* = 3.24, *p* = 0.015). Orangutans also showed a tendency to outperform gorillas (*z* = 2.68, *p* = 0.080). The other observed differences between species were not significant after correcting for multiple comparisons (Table S2). Finally, all species performed below chance in the first ExpSR session (bonobos, spider monkeys and capuchin monkey: *p* < 0.001, gorillas: *p* = 0.007), except for chimpanzees and orangutans, which performed at chance levels.

### Behavioural flexibility (ExpLR and ContSR vs. ContLR)

In Model 2, the comparison between the full model and the null model was significant (GLMM: *χ*^2^ = 462.21, df = 17, *p* < 0.001). The two-way species*trial type interaction was significant (GLMM: *χ*^2^ = 96.79, df = 10, *p* < 0.001; Table 3), indicating inter-specific differences in performance across trial types.

**Table 3:** Results of Models 2 and 3, in which the dependent variable was the selection of LR in ContSR, ExpLR trials and ContLR trials.

**Test Category**	MODEL 2			MODEL 3		
	***χ*** ^**2**^	**Df**	***P***	***χ*** ^**2**^	**Df**	***P***
**Species × Trial type**	**96.79**	**10**	**<0.001**	**75.90**	**10**	**<0.001**
Session	0.12	1	0.729	0.24	1	0.624
Subject’s sex	0.23	1	0.629	0.09	1	0.766
Subject’s age class	1.59	2	0.451	0.12	2	0.940
Partner’s position	0.14	1	0.711	0.06	1	0.808
**Performance in Preference phase**	**7.51**	**1**	**0.006**	**3.91**	**1**	**0.048**
Trial number	0.68	1	0.409	0.30	1	0.582

Significant results in bold. See text for details on the test and control predictors.

Overall, all species performed at ceiling in ExpLR (lowest probability of correct response: 0.99) and ContLR (0.95) trials. In fact, there were no inter-specific differences in ContLR nor ExpLR performance (Tables S3; Fig. S1). In ContSR trials, however, orangutans (0.97; *z* = 3.51, *p* = 0.006), spider monkeys (0.97; *z* = 3.70, *p* = 0.003), and gorillas (0.96; *z* = 3.00, *p* = 0.032) all significantly outperformed bonobos (0.76). Moreover, both orangutans (*z* = 2.73, *p* = 0.070) and spider monkeys (*z* = 2.79, *p* = 0.060) showed a trend towards outperforming capuchin monkeys (0.86).

Post-hoc tests further revealed that most species performed worse in ContSR than in ContLR trials (chimpanzees: *z* = 6.59, *p* < 0.001, bonobos: *z* = 6.99, *p* < 0.001, gorillas: *z* = 4.18, *p* < 0.001, capuchin monkeys: *z* = 7.34, *p* < 0.001). In contrast, orangutans (*z* = 1.64, *p* = 0.228) and spider monkeys’ performance (*z* = 1.79, *p* = 0.175) in ContSR trials was statistically indistinguishable from that in ContLR trials (Table S4). Performance was similarly high in ExpLR and ContLR trials in all species (Table S4), although gorillas (*z* = 3.21, *p* = 0.004) and spider monkeys (*z* = 3.50, *p* = 0.001) performed better in ExpLR than in ContLR trials.

Model 3, which only included sessions in which subjects inhibited choice of the LR in at least 50% of ExpSR trials, yielded similar results to those of Model 2. The comparison between the full model and the null model was significant (GLMM: *χ*^2^ = 501.17, df = 17, *p* < 0.001). The two-way species*trial type interaction was significant (GLMM: *χ*^2^ = 75.90, df = 10, *p* < 0.001; Table 3).

**Figure 3:**
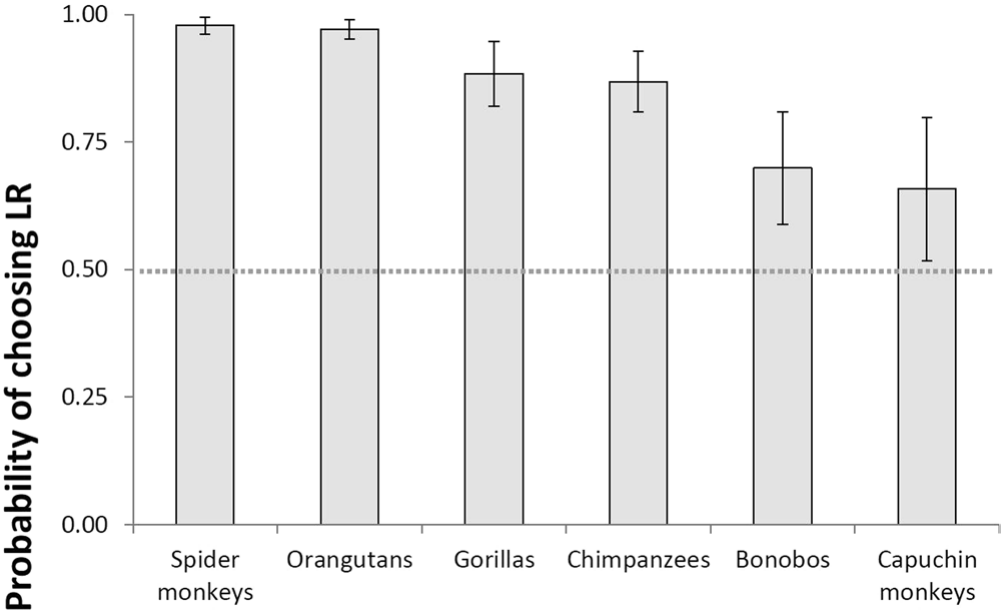
Mean (±SE) probability of making the correct choice (LR) in all ContSR sessions for each species. Results are averaged over the levels of subject’s sex and age, and partner’s position (Model 3). The dotted line illustrates the probability of making a correct choice by chance. Mean probabilities in ExpLR sessions are not reported, as there were no inter-specific differences in these sessions.

Overall, all species performed at ceiling in ExpLR (lowest probability of correct response: 1.0) and ContLR (0.94) trials. There were no significant differences in ContLR nor ExpLR performance across species (Table S3), except that chimpanzees showed a trend towards outperforming spider monkeys in ContLR (*z* = −2.80, *p* = 0.057). In ContSR trials, both orangutans (0.97) and spider monkeys (0.98) significantly outperformed bonobos (0.70; vs. orangutans: *z* = −3.50, *p* = 0.006; vs. spider monkeys: *z* = −3.41, *p* = 0.009) and capuchin monkeys (0.66; vs. orangutans: *z* = −3.52, *p* = 0.006; vs. spider monkeys: *z* = −3.34, *p* = 0.011; Fig. 3).

Post-hoc tests revealed that most species performed worse in ContSR than in ContLR trials (orangutans: *z* = 3.27, *p* = 0.003, chimpanzees: *z* = 6.48, *p* < 0.001, bonobos: *z* = 6.64, *p* < 0.001, gorillas: *z* = 4.37, *p* < 0.001, capuchin monkeys: *z* = 6.87, *p* < 0.001), except for spider monkeys, whose performance in ContSR trials was similar to that in ContLR trials (*z* = −1.70, *p* = 0.204; Table S4). Performance was similarly high in ExpLR and ContLR trials in all species (Table S4), although gorillas performed better in ExpLR than in ContLR trials (*z* = 2.70, *p* = 0.019). A similar trend emerged for spider monkeys (*z* = 2.28, *p* = 0.059).

## Discussion

In this study, we compared for the first time behavioural flexibility and social inhibitory skills in great apes and monkeys by using the same intuitive experimental setup. Depending on the social context (i.e. the presence and position of a conspecific partner), subjects had to flexibly inhibit their preference for a larger reward (LR) in order to maximize their food intake. Overall, our study provided evidence that inhibitory skills and behavioural flexibility are partly independent phenomena, and that both vary across species (Table 1). In particular, orangutans and chimpanzees showed the highest levels of inhibitory skills, going for the smaller reward (SR) when needed (ExpSR trials). Bonobos and spider monkeys did not differ from the other species in terms of inhibition. Finally, gorillas and especially capuchin monkeys were worse than some other species at inhibiting the selection of LR in ExpSR trials. In terms of behavioural flexibility, orangutans and spiders monkeys were the best performers, as indicated by their ability to successfully switch their strategy across trial types and select LR in ContSR and ExpLR trials, when generalizing the inhibition of LR would have been the incorrect response. While chimpanzees and gorillas did not differ from the other species in terms of behavioural flexibility, bonobos and capuchin monkeys performed worse than other species.

Overall, species exhibited different degrees of inhibiting their preference for the selection of LR when required to do so to obtain a food reward (i.e. in ExpSR trials). Chimpanzees and especially orangutans were more successful than the other species, inhibiting the selection of LR in ExpSR trials from the very first session. Orangutans’ exceptional performance is in line with other findings showing very high levels of inhibition in this species^53–55^. Bonobos and spider monkeys, in contrast, did not differ from the other species in terms of inhibition. Finally, capuchin monkeys and to a lesser extent gorillas inhibited less than the other species. Phylogenetic relatedness failed to predict performance in ExpSR trials: despite the fact that most great apes performed relatively well, spider monkeys showed ability to inhibit similar to that of bonobos, and gorillas failed to perform as well as the other apes. Degree of frugivorousness also failed to predict the distribution of inhibitory skills, as the other species failed to outperform gorillas, the most folivorous species, in ExpSR trials. In line with their dietary breadth, orangutans outperformed all other species; however, if dietary breadth played a major role in inhibition, chimpanzees, gorillas and capuchin monkeys should have outperformed bonobos and spider monkeys, which was not the case.

Overall, our results seemed to better reflect the degree of fission-fusion dynamics (see Introduction), with capuchin monkeys and to a lesser extent gorillas being outperformed by two species with high levels of fission-fusion dynamics, and spider monkeys performing like bonobos in ExpSR trials. However, spider monkeys and bonobos failed to significantly outperform gorillas and capuchin monkeys, and only orangutans, but not chimpanzees, tended to perform significantly better than gorillas. Therefore, our results on social inhibition only partially match our previous findings on physical inhibition, in which species with a higher degree of fission-fusion dynamics (i.e. chimpanzees, bonobos, orangutans and spider monkeys) clearly outperformed species living in more cohesive groups (i.e. gorillas, capuchin monkeys and long-tailed macaques^53^). It is noteworthy that the relative distribution of inhibitory skills across species in the social domain found in this study was identical to the one in Amici and colleagues^53^ in the physical domain. Possibly, clearer inter-specific differences in the previous study are simply a result of the previous study having compared performance across five inhibition tasks, while the current study only compared performance in one task.

Behavioural flexibility also varied across species, although orangutans and capuchin monkeys were consistent in being the best and the worse performing species, respectively, like in the distribution of inhibitory skills. Chimpanzees, gorillas and, especially, bonobos and capuchin monkeys performed significantly worse in ContSR trials compared to ContLR trials. This pattern held for the subset of sessions in which subjects inhibited the selection of LR in at least 50% of the ExpSR trials. These results indicate that species other than orangutans and spider monkeys did not promptly switch strategy across types of trials. In particular, these species generalized the selection of SR from ExpSR trials to ContSR trials, showing limited behavioural flexibility, which resulted in reduced food intake. In contrast, spider monkeys and orangutans showed higher behavioural flexibility, selecting SR in ExpSR trials but switching to LR across the other trial types, including ContSR. These results suggest that some species, despite being able to successfully inhibit in ExpSR trials, may have difficulty in flexibly switching strategy depending on the social context. Similarly, flexible behaviour may be present in species showing limited inhibitory skills.

These results confirm and expand those by Amici and colleagues^34^ on tactical deception, in which the species that were the best at inhibiting food retrieval were not necessarily the most successful at solving the task and thus having the highest food intake. In the current study, two species with a high degree of fission-fusion dynamics (i.e. orangutans and spider monkeys) were more efficient than the other species when they had to assess the social context and *switch* strategy depending on the position of the food and the presence of a partner. Importantly, this study provides a new paradigm to test both behavioural flexibility and social inhibition, allowing differentiating the two abilities in the social domain. It is interesting to further note that performance differences across species only emerged in ContSR, but not in ExpLR trials (as shown by chimpanzees, bonobos, gorillas and capuchin monkeys performing worse in ContSR than in ContLR trials). Given that ContSR and ExpSR shared an identical LR (in contrast to ExpLR and ContLR trials, sharing an identical partner location), it is likely that subjects relied more on food rather than partner location when making their choices, generalizing strategies across trial types with a similar food (but not partner) distribution.

Overall, our study provided evidence that inhibitory skills and behavioural flexibility are only partially overlapping abilities, which both vary across species. These conclusions, however, need to be taken with caution. First of all, as with most studies in comparative cognition, our sample size for each species was relatively small. It is therefore not possible to exclude that inter-individual differences might be partly responsible for the inter-specific differences we found. This is especially problematic in studies on behavioural flexibility, as the ability to flexibly solve novel problems is known to differ across individuals of the same species^7^, possibly depending on temperamental differences. In addition, future studies should control for the quality of the social relationship of each tested dyad. Although we have no reason to expect that individuals with a better relationship to their testing partner perform worse in terms of inhibition and/or behavioural flexibility, it may be possible (although unlikely^56^) that subjects with a better relationship are more willing to prosocially share food and thus are less successful when it comes to social inhibition. Secondly, inhibitory control might be highly context-dependent^57–59^. Capuchin monkeys, for example, performed rather poorly in this study, but they are nonetheless known to perform relatively well in delay of gratification tasks, which also require inhibitory skills^53,60–62^. Therefore, the comparative study of inhibition needs to include a wide range of problem-solving contexts, both in the physical and social domain, to ascertain how inhibitory skills are distributed across species, and how they are linked to behavioural flexibility. Nonetheless, our study shows that the ability to inhibit and to flexibly switch strategies depending on the social context may be linked in more complex ways than usually thought, although both abilities may play a crucial role in efficiently solving novel problems.

## Electronic supplementary material

Supplementary Material 1

Orangutans - Preference phase

Bonobos - Training phase

Gorillas - Experimental condition

Spider monkeys - Experimental condition

Capuchin monkeys - Control condition

Chimpanzees - Control condition

Dataset 1
