# Supplementary material for: Social inhibition and behavioural flexibility when the context changes: a comparison across six primate species
Source: Sci Rep. 2018 Feb 15;8:3067. doi: 10.1038/s41598-018-21496-6 (PMC5814526; doi:10.1038/s41598-018-21496-6)
Supplement: Supplementary file 1 — Supplementary Material 1 [file 41598_2018_21496_MOESM1_ESM.pdf]

TITLE PAGE

Title: Social inhibition and behavioural flexibility when the context changes: a comparison across six primate species.

Authors' names: Federica Amici [1,2,*], Josep Call [3,4], Julia Watzek [5], Sarah Brosnan [5,6], Filippo Aureli [7,8]

Affiliations:

[1] Institute of Biology, Faculty of Life Sciences, University of Leipzig, 04103 Leipzig, Germany

[2] Jr. Research Group "Primate Kin Selection", Department of Primatology, Max Planck Institute for Evolutionary Anthropology, 04103 Leipzig, Germany

[3] Department of Comparative and Developmental Psychology, Max Planck Institute for Evolutionary Anthropology, 04103 Leipzig, Germany

[4] School of Psychology and Neuroscience, University of St Andrews, St Andrews, KY16 9JP, Scotland, UK

[5] Department of Psychology and Language Research Center, Georgia State University, P.O. Box 5010, Atlanta, GA, USA

[6] Center for Behavioural Neuroscience and Neuroscience Institute, Georgia State University, P.O. Box 5030, Atlanta, GA, USA

[7] Instituto de Neuroetologia, Universidad Veracruzana, 91190 Xalapa, Veracruz, Mexico

[8] Research Centre in Evolutionary Anthropology and Palaeoecology, Liverpool John Moores University, L3 5UA Liverpool, UK

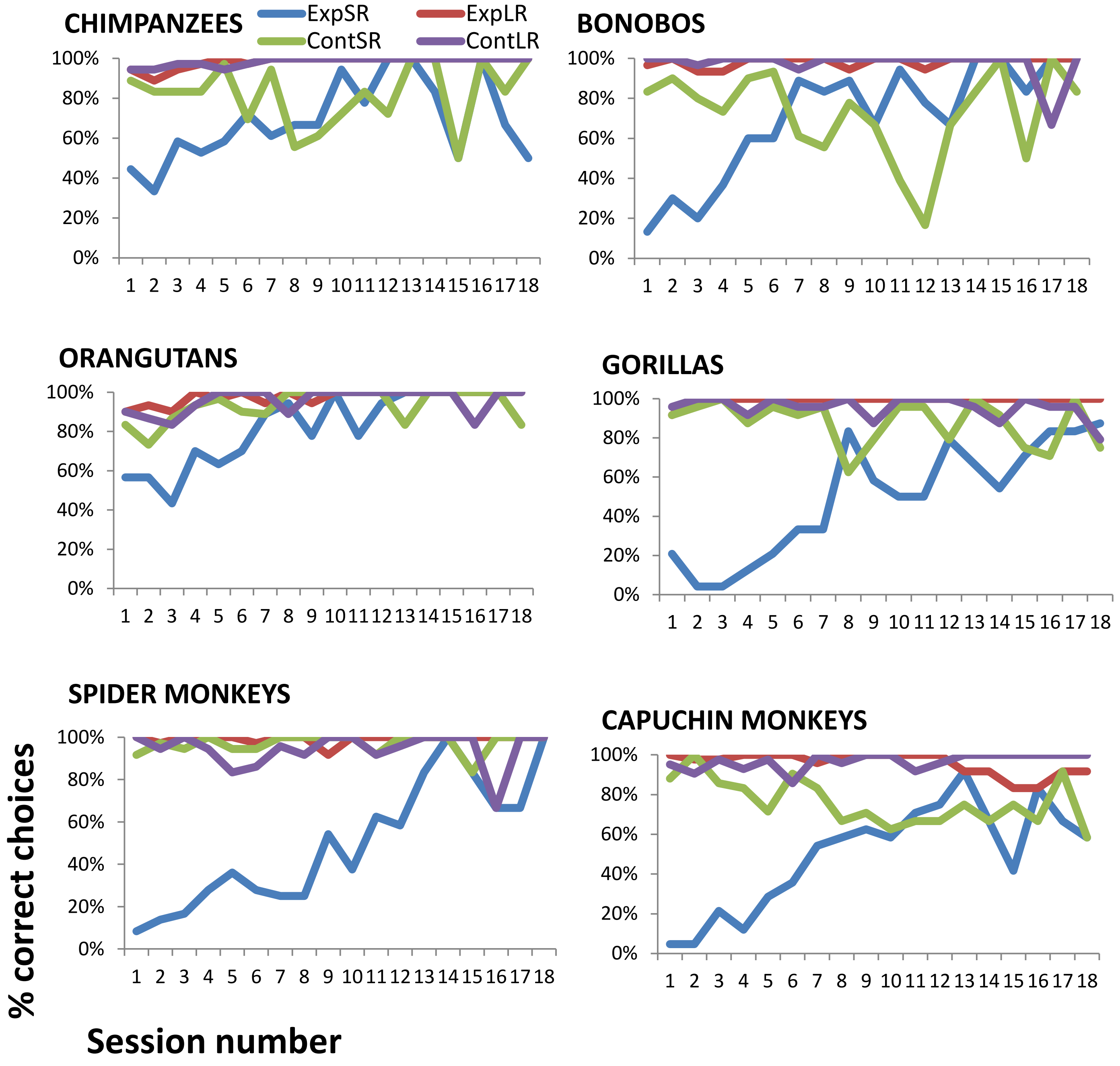

FIGURE S1. For each species and trial type, mean proportion of correct choices per session. Performance in ExpSR trials was used as a measure of inhibition. Performance in ContSR and ExpLR trials (and their comparison to ContLR trials) was used as a measure of behavioural flexibility. Please note how performance in ContSR trials tended to decrease in most species as performance in ExpSR reached a certain threshold.

TABLE S1. For each species, list of the dyads tested, with the order in which conditions were administered and the number of total partners for each subject.

SPECIES	SUBJECT	PARTNER	ORDER OF CONDITIONS	SUBJECT'S NUMBER OF PARTNERS	KINSHIP
Chimpanzees	Fraukje	Kara	EC	3	Mother-daughter
	Fraukje	Lobo	CE	3	None (5-10 years)
	Fraukje	Sandra	EC	3	None (>10 years)
	Kara	Kofi	CE	1	Paternal siblings
	Kofi	Robert	EC	1	Son-father
	Lobo	Kofi	CE	2	Paternal siblings
	Lobo	Robert	EC	2	Son-father
	Robert	Lobo	CE	1	Father-son
	Sandra	Fraukje	CE	2	None (>10 years)
	Sandra	Kara	EC	2	Paternal siblings
Bonobos	Fimi	Kuno	CE	2	Daughter-father
	Fimi	Luisa	EC	2	None (<5 years)
	Kuno	Yasa	CE	1	None (5-10 years)
	Lexi	Fimi	EC	2	None (<5 years)
	Lexi	Yasa	CE	2	None (<5 years)
	Luisa	Lexi	EC	1	None (<5 years)
	Yasa	Fimi	EC	3	None (5-10 years)
	Yasa	Kuno	EC	3	None (5-10 years)
	Yasa	Lexi	CE	3	None (<5 years)
Gorillas	Abeku	Kibara	EC	3	None (<5 years)
	Abeku	Kumili	CE	3	None (<5 years)
	Abeku	Viringika	CE	3	None (<5 years)
	Kibara	Abeku	EC	3	None (<5 years)
	Kibara	Kumili	CE	3	None (<5 years)

	Kibara	Viringika	CE	3	Daughter-mother
	Kumili	Abeku	EC	3	None (<5 years)
	Kumili	Kibara	CE	3	None (<5 years)
	Kumili	Viringika	EC	3	None (<5 years)
	Viringika	Abeku	CE	3	None (<5 years)
	Viringika	Kibara	EC	3	Mother-daughter
	Viringika	Kumili	EC	3	None (<5 years)
Orangutans	Bimbo	Padana	CE	1	None (>10 years)
	Dokana	Bimbo	EC	3	None (>10 years)
	Dokana	Padana	EC	3	None (>10 years)
	Dokana	Pini	CE	3	None (>10 years)
	Padana	Dokana	CE	2	None (>10 years)
	Padana	Raja	EC	2	Maternal paternal siblings
	Pini	Bimbo	CE	2	None (>10 years)
	Pini	Raja	EC	2	Mother-daughter
	Raja	Pini	EC	1	Daughter-mother
Spider monkeys	Capoccetta	Coco	EC	3	None (>10 years)
	Capoccetta	PeloBlanco	EC	3	None (<5 years)
	Capoccetta	RombaGrande	EC	3	None (>10 years)
	Chavito	Coco	CE	1	None (>10 years)
	Coco	Chavito	EC	1	None (>10 years)
	PeloBlanco	Chavito	CE	2	None (<5 years)
	PeloBlanco	Coco	EC	2	None (<5 years)
	RombaGrande	Chavito	CE	2	None (>10 years)
	RombaGrande	Coco	EC	2	None (>10 years)
	RombaPequena	Coco	CE	2	None (<5 years)
	RombaPequena	PeloBlanco	EC	2	None (<5 years)
Capuchin monkeys	Gabe	Nkima	CE	1	None (5-10 years)
	Liam	Gabe	EC	3	None (5-10 years)

	Liam	Gambit	CE	3	None (5-10 years)
	Liam	Logan	CE	3	Maternal siblings
	Lily	Griffin	CE	1	None (5-10 years)
	Logan	Gabe	CE	3	None (5-10 years)
	Logan	Gambit	EC	3	None (5-10 years)
	Logan	Liam	EC	3	Maternal siblings
	Nala	Gabe	CE	2	None (5-10 years)
	Nala	Gambit	EC	2	None (5-10 years)
	Nkima	Gabe	EC	2	None (5-10 years)
	Nkima	Gambit	CE	2	None (5-10 years)
	Wren	Lily	EC	1	None (> 10 years)

In Kinship we report whether the individuals in the dyad are maternal/paternal siblings (for capuchins, paternal relatedness is unknown), mother/father-offspring, or non-kin (specifying how many years they had lived in the same enclosure at the moment of testing).

TABLE S2. Post-hoc tests revealing differences across species in Model 1. Estimates, SE, z and p values are included for each test.

**Contrast**	**Estimate**	**SE**	***z***	***p***
Orangutans - Chimpanzees	0.15	0.77	0.19	1.000
Orangutans - Bonobos	1.18	0.79	1.49	0.673
Orangutans - Spider Monkeys	1.77	0.75	2.36	0.172
Orangutans - Gorillas	*2.18*	*0.81*	*2.68*	*0.080*
**Orangutans - Capuchins**	**2.60**	**0.76**	**3.43**	**0.008**
Chimpanzees - Bonobos	1.04	0.73	1.42	0.716
Chimpanzees - Spider Monkeys	1.62	0.74	2.19	0.244
Chimpanzees - Gorillas	2.03	0.87	2.35	0.174
**Chimpanzees - Capuchins**	**2.45**	**0.76**	**3.24**	**0.015**
Bonobos - Spider Monkeys	0.59	0.75	0.79	0.970
Bonobos - Gorillas	1.00	0.90	1.12	0.875
Bonobos - Capuchins	1.42	0.79	1.79	0.470
Spider Monkeys - Gorillas	0.41	0.83	0.50	0.996
Spider Monkeys - Capuchins	0.83	0.75	1.10	0.880
Gorillas - Capuchins	0.42	0.88	0.47	0.997

Significant results in bold, trends in italics.

TABLE S3. For each trial type, post-hoc tests revealing differences across species in Models 2 and 3. Estimates, SE, *z* and *p* values are included for each significant test.

		Model 2				Model 3			
**Trial Type**	**Contrast**	**Estimate**	**SE**	***z***	***p***	**Estimate**	**SE**	***z***	***p***
ExpLR	Orangutans - Spider Monkeys	-0.31	0.81	-0.38	0.999	0.49	1.07	0.46	0.997
	Orangutans - Gorillas	-2.23	0.98	-2.29	0.200	-1.17	1.30	-0.90	0.947
	Orangutans - Chimpanzees	0.02	0.72	0.02	1.000	-1.18	1.10	-1.08	0.890
	Orangutans - Capuchins	0.06	0.70	0.09	1.000	0.85	1.01	0.84	0.960
	Orangutans - Bonobos	0.19	0.78	0.25	1.000	-0.17	1.09	-0.16	1.000
	Spider Monkeys - Gorillas	-1.93	1.04	-1.85	0.435	-1.67	1.41	-1.18	0.847
	Spider Monkeys - Chimpanzees	0.32	0.80	0.40	0.999	-1.68	1.23	-1.36	0.752
	Spider Monkeys - Capuchins	0.37	0.79	0.46	0.997	0.35	1.17	0.30	1.000
	Spider Monkeys - Bonobos	0.50	0.84	0.59	0.992	-0.67	1.22	-0.55	0.994
	Gorillas - Chimpanzees	2.25	0.98	2.30	0.196	-0.01	1.42	-0.01	1.000
	Gorillas - Capuchins	2.29	0.97	2.35	0.173	2.02	1.35	1.49	0.671
	Gorillas - Bonobos	2.43	1.02	2.37	0.166	1.00	1.43	0.70	0.982
	Chimpanzees - Bonobos	0.18	0.74	0.24	1.000	1.01	1.22	0.83	0.962
	Chimpanzees - Capuchins	0.05	0.69	0.07	1.000	2.03	1.14	1.78	0.481

	Capuchins - Bonobos	0.13	0.76	0.18	1.000	-1.02	1.17	-0.87	0.953
ContLR	Orangutans - Spider Monkeys	1.05	0.66	1.59	0.606	2.06	0.88	2.35	0.174
	Orangutans - Gorillas	-0.37	0.70	-0.53	0.995	1.42	0.89	1.60	0.596
	Orangutans - Chimpanzees	-0.59	0.71	-0.83	0.962	-0.61	0.96	-0.63	0.989
	Orangutans - Capuchins	0.26	0.65	0.41	0.999	1.11	0.96	1.16	0.854
	Orangutans - Bonobos	-0.88	0.83	-1.06	0.896	0.38	0.96	0.39	0.999
	Spider Monkeys - Gorillas	-1.43	0.68	-2.09	0.295	-0.64	0.86	-0.74	0.977
	Spider Monkeys - Chimpanzees	-1.64	0.68	-2.40	0.155	*-2.67*	*0.95*	*-2.80*	*0.057*
	Spider Monkeys - Capuchins	-0.79	0.62	-1.27	0.804	-0.95	0.96	-0.99	0.922
	Spider Monkeys - Bonobos	-1.93	0.79	-2.45	0.139	-1.68	0.94	-1.80	0.469
	Gorillas - Chimpanzees	-0.21	0.74	-0.29	1.000	-2.03	0.93	-2.18	0.246
	Gorillas - Capuchins	0.64	0.69	0.92	0.942	-0.31	0.93	-0.33	1.000
	Gorillas - Bonobos	-0.51	0.85	-0.60	0.991	-1.04	0.95	-1.10	0.882
	Chimpanzees - Capuchins	0.85	0.67	1.28	0.798	1.73	0.99	1.75	0.500
	Chimpanzees - Bonobos	-0.30	0.82	-0.36	0.999	0.99	0.99	1.00	0.918
	Capuchins - Bonobos	-1.14	0.80	-1.43	0.709	-0.74	1.02	-0.72	0.979
ContSR	Orangutans - Spider Monkeys	-0.10	0.67	-0.14	1.000	-0.32	0.89	-0.36	0.999
	Orangutans - Gorillas	0.27	0.66	0.41	0.999	1.47	0.79	1.86	0.425
	Orangutans - Chimpanzees	1.37	0.61	2.25	0.215	1.62	0.75	2.17	0.253
	Orangutans - Capuchins	*1.62*	*0.60*	*2.73*	*0.070*	**2.85**	**0.81**	**3.52**	**0.006**

Orangutans - Bonobos	**2.28**	**0.65**	**3.51**	**0.006**	**2.66**	**0.76**	**3.50**	**0.006**
Spider Monkeys - Gorillas	0.36	0.68	0.54	0.995	1.79	0.91	1.97	0.361
Spider Monkeys - Chimpanzees	1.47	0.63	2.35	0.174	1.94	0.88	2.21	0.235
Spider Monkeys - Capuchins	*1.72*	*0.62*	*2.79*	*0.060*	**3.17**	**0.95**	**3.34**	**0.011**
Spider Monkeys - Bonobos	**2.37**	**0.64**	**3.70**	**0.003**	**2.98**	**0.88**	**3.41**	**0.009**
Gorillas - Chimpanzees	1.11	0.64	1.74	0.506	0.14	0.74	0.20	1.000
Gorillas - Capuchins	1.35	0.64	2.13	0.270	1.37	0.81	1.70	0.534
Gorillas - Bonobos	**2.01**	**0.67**	**3.00**	**0.032**	1.19	0.77	1.54	0.639
Chimpanzees - Capuchins	0.25	0.55	0.45	0.998	1.23	0.74	1.65	0.563
Chimpanzees - Bonobos	0.90	0.57	1.59	0.608	1.04	0.68	1.54	0.642
Capuchins - Bonobos	0.65	0.60	1.10	0.884	-0.19	0.80	-0.23	1.000

Significant results in bold, trends in italics.

TABLE S4. For each species, post-hoc tests revealing significant differences of interest across trial types in Models 2 and 3. Estimates, SE, z and p values are included for each significant test.

		**Model 2**				**Model 3**			
**Species**	**Contrast**	**Estimate**	**SE**	***z***	***p***	**Estimate**	**SE**	***z***	***p***
Orangutans	ContLR - ContSR	0.53	0.32	1.64	0.228	**1.38**	**0.42**	**3.27**	**0.003**
	ExpLR - ContLR	0.49	0.40	1.23	0.433	0.16	0.53	0.30	0.952
Spider monkeys	ContLR - ContSR	0.62	0.35	1.79	0.175	-1.00	0.59	-1.70	0.204
	ExpLR - ContLR	**1.85**	**0.53**	**3.50**	**0.001**	*1.72*	*0.75*	*2.28*	*0.059*
Gorillas	ContLR - ContSR	**1.17**	**0.28**	**4.18**	**<.001**	**1.44**	**0.33**	**4.37**	**<.001**
	ExpLR - ContLR	**2.35**	**0.73**	**3.21**	**0.004**	**2.75**	**1.02**	**2.70**	**0.019**
Chimpanzees	ContLR - ContSR	**2.49**	**0.38**	**6.59**	**<.001**	**3.61**	**0.56**	**6.48**	**<.001**
	ExpLR - ContLR	0.11	0.47	0.24	0.969	0.73	0.87	0.83	0.683
Capuchin monkeys	ContLR - ContSR	**1.89**	**0.26**	**7.34**	**<.001**	**3.12**	**0.45**	**6.87**	**<.001**
	ExpLR - ContLR	0.69	0.38	1.83	0.161	0.42	0.65	0.65	0.794
Bonobos	ContLR - ContSR	**3.69**	**0.53**	**6.99**	**<.001**	**3.67**	**0.55**	**6.64**	**<.001**
	ExpLR - ContLR	0.59	0.63	0.93	0.622	0.71	0.87	0.81	0.697

Significant results in bold, trends in italics.
